# Bionomics and insecticide resistance of *Aedes albopictus* in Shandong, a high latitude and high-risk dengue transmission area in China

**DOI:** 10.1186/s13071-020-3880-2

**Published:** 2020-01-09

**Authors:** Hongmei Liu, Luhong Liu, Peng Cheng, Linlin Yang, Junhu Chen, Yao Lu, Haifang Wang, Xiao-Guang Chen, Maoqing Gong

**Affiliations:** 1 Department of Pathogen Biology, Guangdong Provincial Key Laboratory of Tropical Disease Research, School of Public Health, Southern Medical University, Guangzhou, People’s Republic of China; 2 Shandong Institute of Parasitic Diseases, Shandong First Medical University & Shandong Academy of Medical Sciences, Jining, 272033 Shandong People’s Republic of China; 3 Jining Center for Disease Control and Prevention, Jining, 272033 Shandong People’s Republic of China; 4 Guangdong Provincial Institute of Biological Products and Materia Medica, Guangzhou, 510440 People’s Republic of China

**Keywords:** *Aedes albopictus*, Dengue fever, BI, *kdr*, Surveillance

## Abstract

**Background:**

Dengue fever outbreaks tend to spread northward in China, and Jining is the northernmost region where local dengue fever cases have been detected. Therefore, it is important to investigate the density of *Aedes albopictus* and its resistance to deltamethrin.

**Methods:**

The Breteau index (BI) and container index (CI) were calculated to assess the larval density of *Ae. albopictus* and human-baited double net trap (HDN) surveillance was performed in six subordinate counties (Rencheng, Yanzhou, Sishui, Liangshan, Zoucheng and Jiaxiang) of Jining City in 2017 and 2018. The resistance of *Ae. albopictus* adults to deltamethrin was evaluated using the World Health Organization (WHO) standard resistance bioassay. The mutations at *Vgsc* codons 1532 and 1534 were also analysed to determine the association between *kdr* mutations and phenotypic resistance in adult mosquitoes.

**Results:**

The average BI, CI and biting rate at Jining were 45.30, 16.02 and 1.97 (female /man/hour) in 2017 and 15.95, 7.86 and 0.59 f/m/h in 2018, respectively. In August 26, 2017, when the first dengue fever case was diagnosed, the BI at Qianli village in Jiaxiang County was 107.27. The application of prevention and control measures by the government sharply decreased the BI to a value of 4.95 in September 3, 2017. The mortality of field-collected *Ae. albopictus* females from Jiaxiang was 41.98%. I1532T, F1534L and F1534S mutations were found in domain III of the *Vgsc* gene. This study provides the first demonstration that both I1532T and F1534S mutations are positively correlated with the deltamethrin-resistant phenotype.

**Conclusions:**

Mosquito density surveillance, resistance monitoring and risk assessment should be strengthened in areas at risk for dengue to ensure the sustainable control of *Ae. albopictus* and thus the prevention and control of dengue transmission.
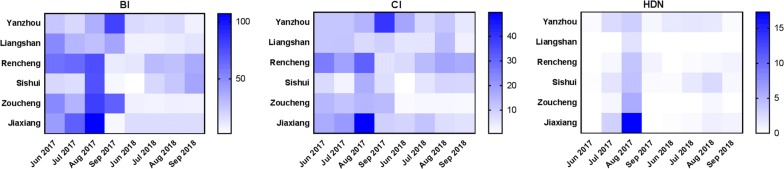

## Background

*Aedes albopictus*, also known as the Asian tiger mosquito, can spread dengue fever [1], chikungunya fever [2] and Zika virus [3, 4] and has become one of the most invasive species in the world due to its strong ability to adapt to new environments [5]. China is undergoing rapid urbanization, which might lead to the emergence and spread of dengue fever due to urban population growth, crowded housing conditions and under-developed waste management systems [6].

**Fig. 1:**
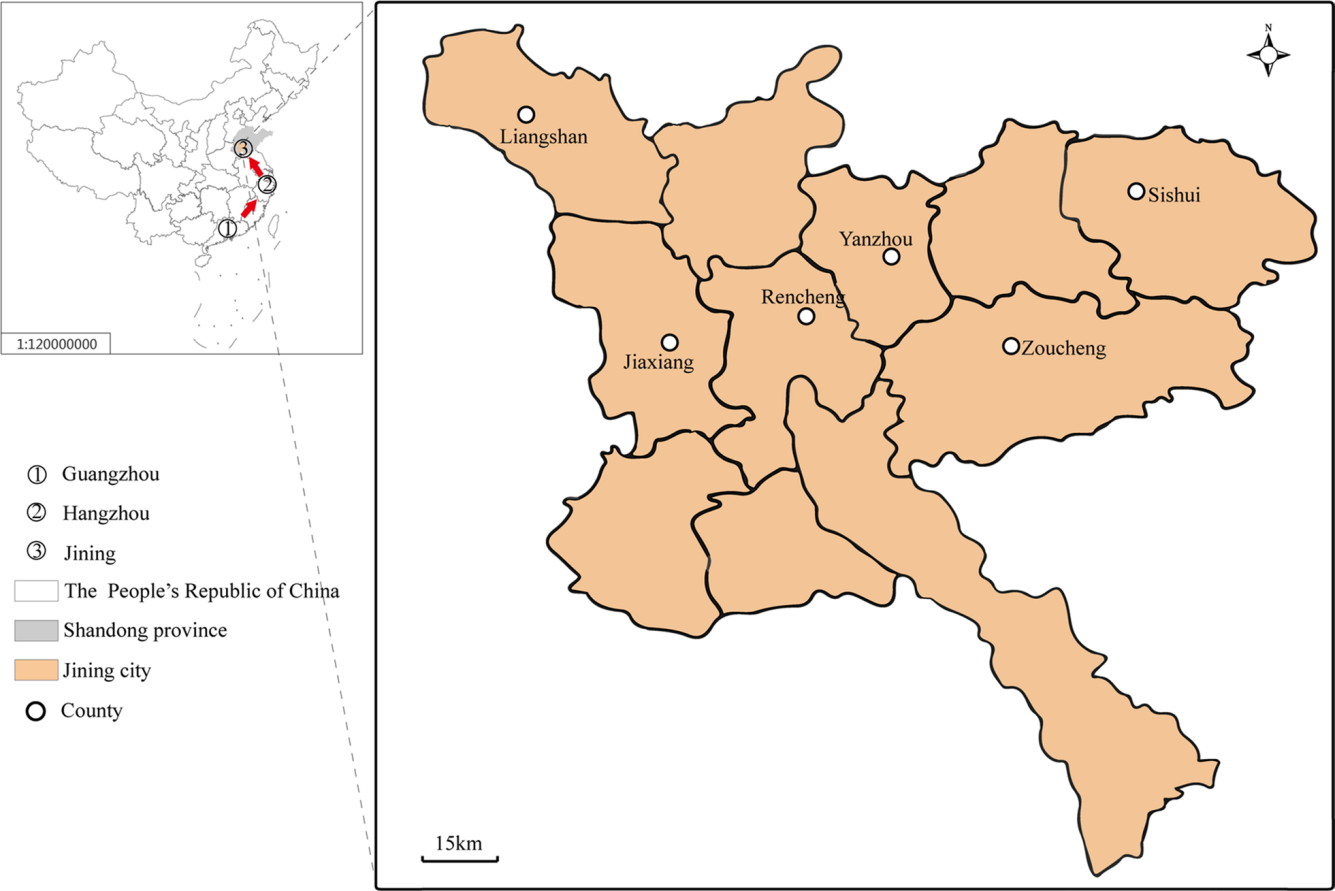
Map of mosquito sample sites in Jining, China

In 2014, more than 40,000 cases of dengue fever, which is spread by *Ae. albopictus*, were reported in Guangzhou [7, 8], and the first local dengue fever case was reported in Hangzhou in 2015 [9] (Fig. 1). Shandong Province reported an autochthonous dengue case for the first time on 16 August 2017. Ninety-five cases were subsequently reported in the whole province, and 79 indigenous cases occurred in Jiaxiang, Jining City [10]. It is obvious that local cases of dengue tend to spread northward. The increase in dengue fever outbreaks in China observed in recent years is concerning. Unfortunately, considering the ability of *Ae. albopictus* to invade new habitats, the increasing frequency of dengue epidemics and the mobility of human populations, it is expected that new indigenous dengue fever cases will continue to occur in Shandong, China, which is similar to the situation in Brazil and some European regions, where dengue cases have emerged due to the introduction of dengue-infected people and invasion of *Aedes* mosquitoes [11, 12]. Dengue fever has become an important public health problem in China [13].

Mosquito surveillance has become an important component of integrated vector management programmes aiming to quantify human risk by determining the local vector abundance [14]. Mosquito surveillance mainly focuses on the collection of larvae and adult mosquitoes. The Breteau index (number of positive containers per 100 houses, BI) and the container index (percentage of water-holding containers infested with larvae or pupae, CI) [15, 16] are the most widely used larval indices for *Aedes* surveillance and the forecasting of disease outbreaks [17]. However, it should be noted that larval indices do not respond well to the abundance of adult mosquitoes. Adult sampling can provide more valuable data for the evaluation of dengue risk and adulticiding measures [16]. The human-baited double net trap (HDN) is an efficient method for *Aedes* monitoring. The HDN consists of two box nets; the inner net protects the human-bait, and the outer net is raised off the ground so that mosquitoes lured to the human-bait are collected between the nets by another collector, who is protected by repellent [18]. According to the Chinese vector monitoring programme [19], HDNs were mainly set to collect mosquitoes in the external environments near the residential areas from 16:30 h to 18:30 h [18], and each collection was performed for a duration of 30 min [18]. According to the Chinese Center for Disease Control and Prevention’s (CDC’s) division standard for *Aedes* monitoring [20], a BI < 5 can be considered the threshold for controlling dengue fever and adult mosquito density by the HDN method should be less than 2. However, more evidence is needed to confirm that a BI < 5 is suitable for preventing dengue transmission because this value was derived from yellow fever transmission surveys [15, 21]. In this study, the larval density of *Ae. albopictus* was measured based on the BI and CI, and adult mosquitoes were captured by the HDN method. Furthermore, the extent and intensity of pyrethroid resistance were evaluated through adult resistance surveillance and the assessment of knockdown resistance (*kdr*) mutations.

## Methods

### Study areas

Shandong Province is located on the eastern coast of China, between 34°22ʹ, 38°24ʹN and 114°47ʹ, 122°42ʹE. Shandong has a typical warm temperate climate with concentrated precipitation. In Shandong, rain and heat occur in the same season, and this area has short springs and autumns and long winters and summers. The annual average temperature is 11–14 °C, and the annual average precipitation is between 550 and 950 mm. However, the precipitation distribution is very uneven, with 60–70% of the annual precipitation concentrated in the summer, and this distribution is suitable for the development and reproduction of *Ae. albopictus*. Jining City, located in the southwestern part of Shandong Province, is dominated by plain depressions, has four national forest parks and is one of the central cities of the Huaihai Economic Zone.

According to the Chinese CDC’s division standard for *Aedes* monitoring [20], Shandong Province can be classified as a class III region, which indicates that it is an area at risk for dengue fever outbreaks based on recently reported imported dengue cases and the distribution of *Aedes* mosquitoes. Six subordinate counties (Rencheng, Yanzhou, Sishui, Liangshan, Zoucheng and Jiaxiang) of Jining City were included as study areas in 2017 and 2018 (Fig. 1, Additional file 1: Table S1).

### Mosquito collection and species identification

Mosquito surveillance was performed once a month in six subordinate counties (Rencheng, Yanzhou, Sishui, Liangshan, Zoucheng and Jiaxiang) of Jining City from June to September in 2017 and 2018. Each monitoring county selected no less than 100 households in four residential areas in different geographical locations. Other habitats, such as hospitals, parks, and waste collection stations, were subject to local conditions. All small water containers around each selected house were inspected for the presence of *Aedes* larvae or pupae, and the number of containers positive for *Aedes* mosquitoes was recorded. The larvae collected from the containers were identified to the species level or transported to the laboratory and reared to adulthood for species identification. All specimens were identified morphologically using taxonomic keys [22]. Molecular identification was conducted for selected adults and unhatched eggs using PCR with species-specific primers for amplification of the ribosomal internal transcribed spacers (ITS1 and ITS2) and *18S* rDNA regions [23–25].

Sampling was conducted twice a month from June to September in 2017 and 2018. The captured mosquitoes were killed by chloroform and then counted and identified based on morphology [26, 27].

Wild populations of *Ae. albopictus* were collected at Jiaxiang in August 2018. The larvae previously collected from the containers were transported to the laboratory and reared to adulthood under standard conditions of 27 ± 2 °C and 75 ± 10% relative humidity.

### Adult bioassay

The larvae previously collected from the containers were transported to the laboratory and reared to adulthood. 3–5-days-old unfed female adults were tested using the World Health Organization (WHO) adult resistance bioassay.

According to WHO susceptibility testing guidelines [28], 20 to 25 female mosquitoes were exposed to 0.03% deltamethrin insecticide-impregnated papers (test) or carrier oil-treated papers without insecticide (control) for 1 h (School of Biological Sciences, Universiti Sains Malaysia, Penang, Malaysia) and then transferred to recovery tubes and maintained on 10% sucrose solution for 24 h. The number of surviving and dead mosquitoes were recorded, and five replicates were included in the experiment. If the mosquito mortality in the control tubes exceeded 10%, the mortality rates of all treated groups were corrected using Abbott’s formula [28]. In the adult bioassay, the surviving mosquitoes were considered resistant, whereas the dead mosquitoes were considered susceptible [29]. The surviving and dead mosquitoes were separately stored in 95% ethanol for subsequent *kdr* mutation genotyping.

### Genotyping of *kdr* mutations

Genomic DNA from individual mosquitoes was extracted using a DNeasy Blood and Tissue Kit (Qiagen, Hilden, Germany) following the manufacturer’s recommended protocol. The molecular identification of *Ae. albopictus* was conducted using the species-specific primers aegSCF7 (5ʹ-GAG AAC TCG CCG ATG AAC TT-3ʹ) and aegSCR7 (5ʹ-GAC GAC GAA ATC GAA CAG GT-3ʹ) [30] to amplify the domain III region of the *Ae. albopictus* para-sodium channel gene α subunit. The PCR products were directly sequenced using Tks Gflex™ DNA Polymerase (Takara Bio, Inc., Shiga, Japan) following the manufacturer’s recommended protocol. All resistant and susceptible individuals were genotyped to determine the presence of *kdr* mutations at codons 1532 and 1534 by direct sequencing at Invitrogen Co., Ltd. (Shanghai, China).

### Data analysis

The *Ae. albopictus* biting rate, which was used as the density indicator, was calculated as the mean number of adult female mosquito bites per man per hour (f/m/h) [1, 31, 32]. Studentʼs t-test was used to compare the mosquito density between 2017 and 2018 and the differences in the BIs and CIs. Heterogeneity of variance test assessed using Leveneʼs test. The density of *Ae. aegypti* in different months and regions of the same year was compared with a one-way ANOVA followed by LSD tests (homogeneity of variance: *P* > 0.05) or Dunnett’s T3 tests (homogeneity of variance: *P* < 0.05). The *Ae. albopictus* biting rates among the various months and regions were assessed using the Kruskal-Wallis test, and the *kdr* allele frequencies of the resistant and susceptible groups were calculated. Fisher’s exact tests were performed to assess the association between *kdr* mutations and resistance, and the odds ratio for each *kdr* allele was determined. The statistical analyses were performed using SPSS software (version 19 for Windows, SPSS Inc., Chicago, IL, USA).

According to the *Aedes* mosquito surveillance guidelines of China [20], a BI less than 5 is the threshold for the control of dengue transmission, whereas values of 5–10, 10–20 and greater than 20 indicate risks of transmission, outbreak, and regional epidemic, respectively.

According to WHO guidelines [28], mortality rates in adult resistance bioassays of 98–100%, 90–97% and less than 90% indicate susceptible (S), 90–97% suspected resistant (SR), and resistant (R) populations, respectively.

## Results

### Breteau index

**Fig. 2:**
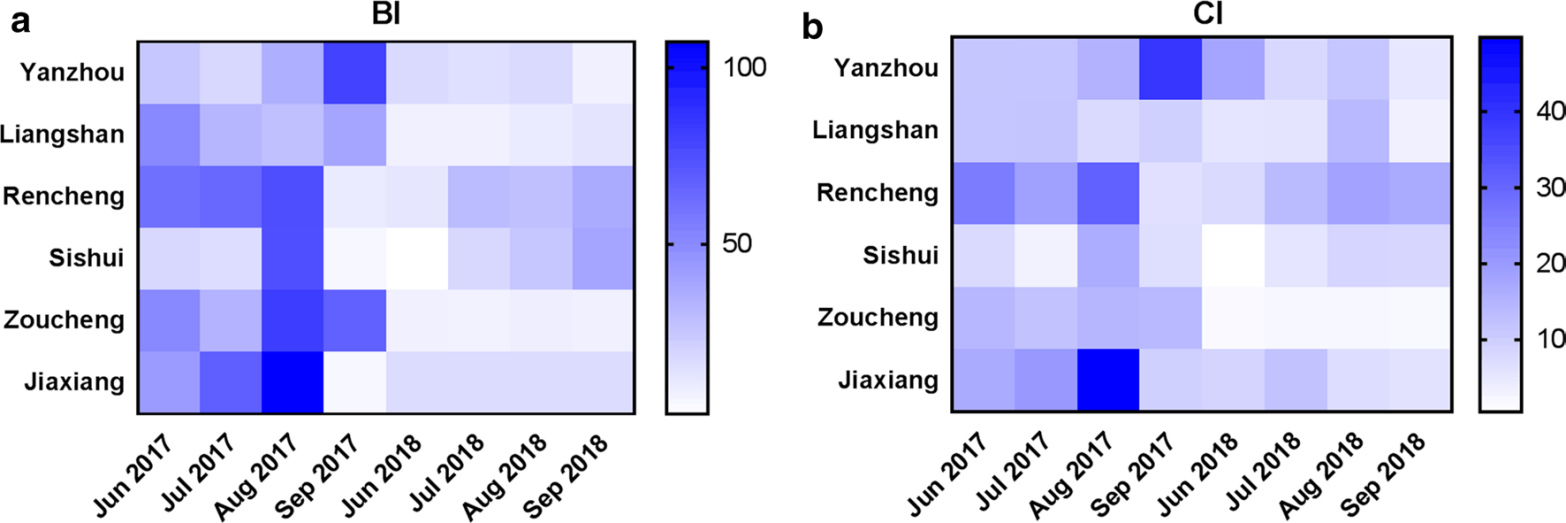
The larval indices (BI and CI) between 2017 and 2018. **a** The BI value between 2017 and 2018. **b** The CI value between 2017 and 2018

The BI in Qianli village in Jiaxiang County was 107.27 in August 2017 (Fig. 2a, Additional file 2: Table S2). A sharp decrease in BI, was observed, reaching 4.95 in September 2017 (Fig. 2a, Additional file 2: Table S2).

**Fig. 3:**
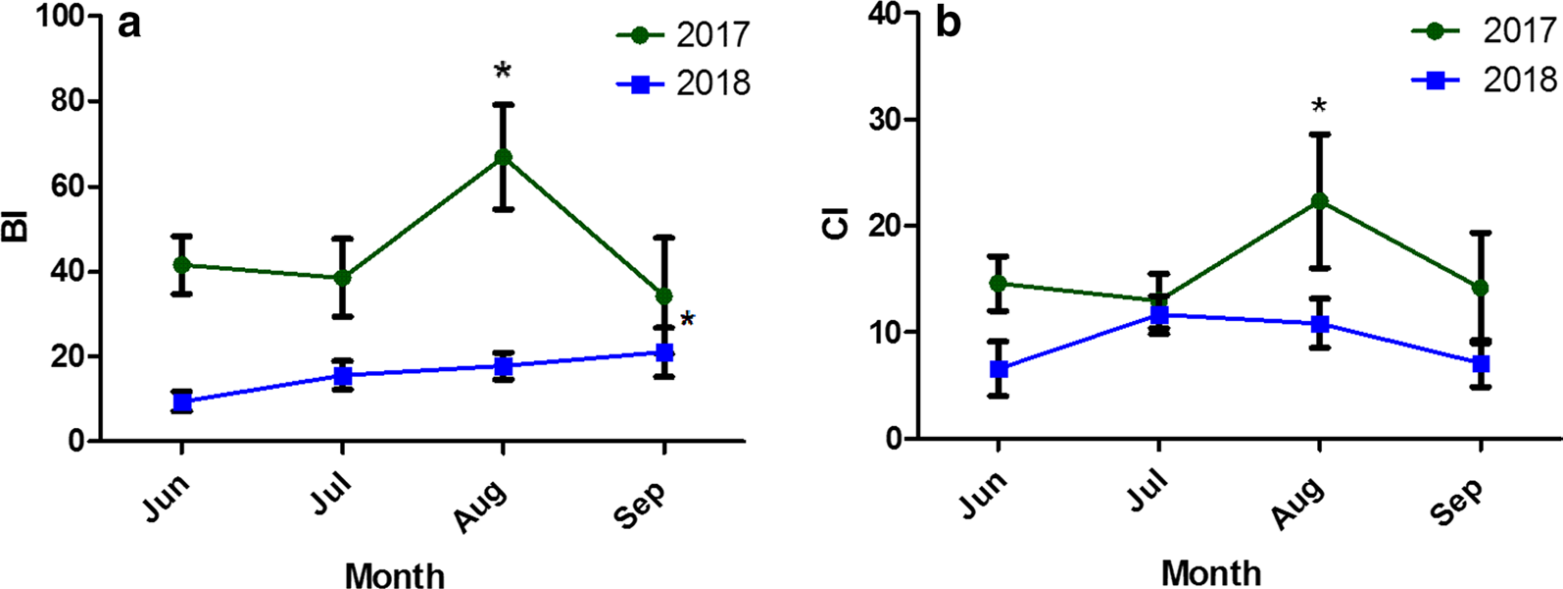
Temporal variation of the larval indices (BI and CI) between 2017 and 2018. **a** Trend in the monthly average BI fluctuation between 2017 and 2018. **b** Trend in the monthly average CI fluctuation between 2017 and 2018

The average BI values for Jining in 2017 and 2018 were 45.30 and 15.95, respectively. The BI value in 2017 was significantly higher than that in 2018 (t-test: *t*_(1426)_ = 43.565, *P* < 0.001). The larval density showed seasonal fluctuations. The highest monthly average BI value for 2017 (BI = 66.88) was obtained in August, and the highest value for 2018 (BI = 17.80) was recorded in August (Fig. 2a, Additional file 2: Table S2). During the survey, the highest BI value (107.27) in Jiaxiang was recorded in August 2017. Levene’s test indicated that all the sample groups showed variance heterogeneity (all *P* < 0.05). The differences in the BI among the various months were assessed by Welch’s (unequal variance) ANOVA (2017: *F*_(3, 538.657)_ = 164.627, *P* < 0.001; 2018: *F*_(3, 208.989)_ = 61.778, *P* < 0.001) (Fig. 3a, Additional file 2: Table S2). It is worth noting that the BI values in August 2017 and September 2018 were higher than those in the other months of the same year, as determined by Dunnett’s T3 test. In addition, a significant difference in BI values was found among the regions (Welch’s ANOVA, 2017: *F*_(5, 450.962)_ = 156.172, *P* < 0.001; 2018: *F*_(5, 120.669)_ = 969.668, *P* < 0.001).

### Container index

The average CI values of Jining in 2017 and 2018 were 16.02 and 7.86, respectively. The CI value for 2018 was clearly lower than that for 2017 (t-test: *t*_(1453)_ = 23.607, *P* < 0.001). The highest CI value (49.72) in Jiaxiang was also recorded in August 2017. The differences in the CI values among the various months were compared by Welch’s ANOVA (2017: *F*_(3, 202.691)_ = 44.276, *P* < 0.001; 2018: *F*_(3, 96.011)_ = 4.742, *P* = 0.004). In 2017, the CI values in August and September were significantly different from other those in the months, whereas in 2018, the CI value in July was significantly different from that in August, as demonstrated by Dunnett’s T3 test. A significant difference in the CI values was also found among the regions (Welch’s ANOVA, 2017: *F*_(5, 164.820)_ = 108.168, *P* < 0.001; 2018: *F*_(5, 76.931)_ = 304.864, *P* < 0.001) (Figs. 2b, 3b, Additional file 3: Table S3).

### Human-baited double net trapping

**Fig. 4:**
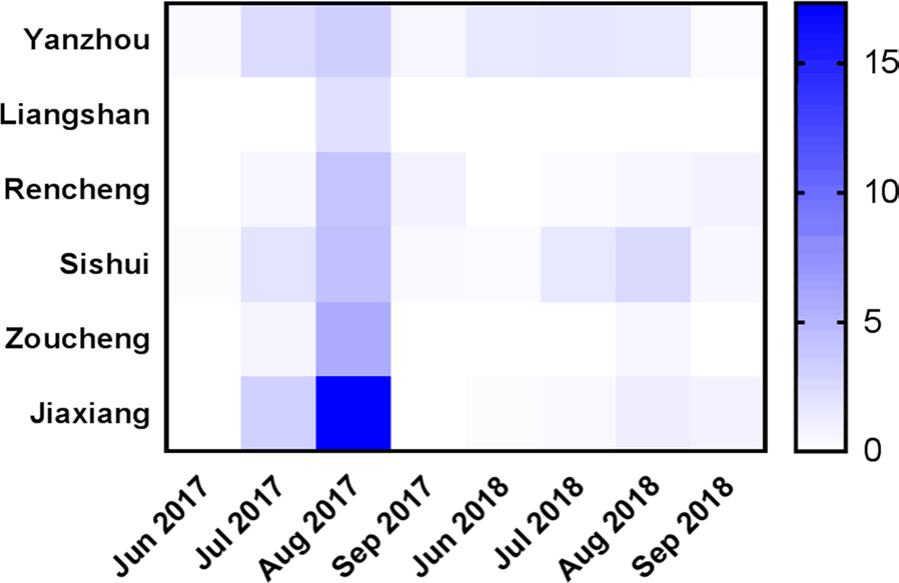
*Aedes albopictus* females collected per person per hour using the HDN method between 2017 and 2018. The biting rate of *Ae. albopictus* was calculated as the mean number of adult female mosquitoes per man per hour (f/m/h)

A total of 215 female *Ae. albopictus* were collected using the HDN technique. The average biting rates were 1.97 f/m/h in 2017 and 0.59 f/m/h in 2018 (Fig. 4, Additional file 4: Table S4). In contrast, the number of collected specimens in 2017 was 3-fold higher than that in 2018, and the difference was statistically significant (t-test: *t*_(49.37)_ = 6.946, *P* < 0.001). In 2017, adult mosquitoes also showed a seasonal preference. The biting rates of *Ae. albopictus* varied significantly among the various months (Kruskal-Wallis test, *χ*^2^ = 23.721, *df* = 2, *P* < 0.0001) and regions (Kruskal-Wallis test, *χ*^2^ = 27.850, *df* = 5, *P* < 0.0001) in 2017, but no significant differences in the biting rates were found among the various months (Kruskal-Wallis test, *χ*^2^ = 5.466, *df* = 3, *P* = 0.141) and regions (Kruskal-Wallis test, *χ*^2^ = 8.596, *df* = 4, *P* = 0.072) in 2018.

### Adult bioassay and *kdr* mutations

The mortality of field-collected female *Ae. albopictus* from Jiaxiang, which is the northernmost location where local dengue fever has been detected in China, was 41.98% (Table 1). Thus, according to the WHO guidelines, the mosquito population of Jiaxiang can be considered an R population. Forty-two susceptible (dead) and 60 resistant (surviving) individuals were reserved for the classification of resistance phenotypes.

**Table 1:** Deltamethrin resistance of *Ae. albopictus* measured using the WHO standard adult bioassay

Sampling site	*n*	Mortality ± SE (%)	Phenotype
Jiaxiang	102	41.98 ± 13.79	R
Laboratory strain	111	99.09 ± 2.03	S

*Abbreviations*: n, number of specimens; R, resistant; S, susceptible; SE, standard error

Codons 1532 and 1534 in domain III of the *Vgsc* (voltage-gated sodium channel) gene exhibited non-synonymous mutations in 102 mosquito individuals from the Jiaxiang population. The wild-type allele ATC/I (93.63%, 191/204) and the mutant allele ACC/T (6.37%, 13/204) were detected at codon 1532, whereas the wild-type allele TTC/F (73.53%, 150/204) and two mutant alleles, TTG/L (19.61%, 40/204), TCC/S (6.86%, 14/204), were detected at codon 1534. However, the mutant allele F1534C was not found in this population. The wild-type genotype I/I (87.25%, 89/102) and the wild-type/mutant heterozygote I/T (12.75%, 13/102) were detected at codon 1532, but the mutant genotype T/T was not detected. A total of six genotypes, including the wild-type genotype F/F (55.88%, 57/102), the wild-type/mutant heterozygotes F/L (30.39%, 31/102) and F/S (4.90%, 5/102), and the mutant genotypes L/L (3.92%, 4/102), S/S (3.92%, 4/102) and L/S (0.98%, 1/102), were detected at codon 1534 (Additional file 5: Table S5).

The I1532T mutant allele at codon 1532 was significantly correlated with deltamethrin resistance (odds ratio = 9.22, *P* = 0.0084) (Table 2). To assess the association between *kdr* mutations at codon 1534 and resistance, the F1534S and F1534L alleles were analysed separately. The F1534S mutation was significantly associated with deltamethrin resistance (odds ratio = 4.56, *P* = 0.0285), but the F1534L mutation did not show significant differences between the R and S groups (odds ratio = 1.21, *P* = 0.3663) (Table 3).

**Table 2:** Association between *kdr* mutations at codon 1532 and phenotypic resistance in *Ae. albopictus*

Phenotype	*n*	Genotype			Odds ratio (95% CI)	*P*-value^a^
1532		II	IT	TT		
S	42	41	1	0	9.22 (1.18–72.36)	0.0084
R	60	48	12	0		

^a^Fisher’s exact probability test

*Abbreviations*: R, resistant; S, susceptible; n, number of samples; II, homozygous isoleucine/ isoleucine; IT, heterozygotes isoleucine/threonine; TT, homozygous threonine/threonine

**Table 3:** Association between *kdr* mutations at codon 1534 and phenotypic resistance in *Ae. albopictus*

Phenotype	*n*	Genotype						Odds ratio (95% CI)		*P*-value^a^	
1534		FF	FL	LL	FS	LS	SS	F1534L	F1534S	F1534L	F1534S
S	42	25	15	0	2	0	0	1.21 (0.55–2.47)	4.55 (0.99–20.92)	0.3663	0.0285
R	60	32	16	4	3	1	4				

^a^Fisher’s exact probability test

*Abbreviations*: R, resistant; S, susceptible; n, number of samples; FF, homozygous phenylalanine/phenylalanine; FL, heterozygotes phenylalanine/leucine, LL, homozygous leucine/ leucine; LS, heterozygotes leucine/serine; SS, homozygous serine/serine

## Discussion

Dengue fever outbreaks tend to spread northward in China, and Jining is the northernmost region where local dengue fever cases had been observed. The present study constitutes the most comprehensive survey (by far) of *Ae. albopictus* mosquitoes in Jining. This study found that the larval indices (BI and CI) and adult density in 2017 were obviously higher than those in 2018. The average BI values in Jining in 2017 and 2018 were 45.30 and 15.95, respectively, and these levels indicate potential risk of dengue outbreak. In addition, this study provides the first demonstration that both the I1532T and F1534S mutations are positively correlated with the deltamethrin resistance phenotype.

The BI is a key tool for determining the risk of dengue transmission [33] and is commonly used for risk assessment and as an early warning system for dengue epidemics [15, 17, 34]. In 2017, Jiaxiang, which had a BI value greater than 100 with reported dengue cases, was at risk of a regional epidemic. After the CDC administered health education programmes, destroyed mosquito populations, and optimized the environment, the BI value decreased sharply to 4.95, and no new dengue fever cases were reported. This finding indicated that placing “anbei” into containers to kill larvae, spraying chemical insecticides, turning over containers, cleaning the water, and administering health education are effective measures for controlling dengue transmission, and these approaches were designed based on the experience in rural mosquito killing. Although the threshold used in this study was not statistically derived, the Chinese CDC states that a BI < 5 constitutes the threshold for the control of dengue transmission. Indeed, in this study, no new cases of dengue fever were recorded after the BI value decreased to less than 5; therefore, it appeared that a BI value < 5 is a reliable threshold value capable of reflecting the incidence of dengue epidemics and facilitating the management of dengue outbreaks. Related research has shown that BI could be used as a predictor of dengue transmission [15, 35], but the specific value of the low-risk BI threshold is controversial. Some studies have suggested that it should be < 1 [15], whereas others have indicated that it should be < 4 [36] or < 5 [14, 20]. Thus, the low-risk BI threshold needs further study.

Notably, the BI values reached 44.75 and 15.95 in 2018 and 2017, respectively, and these levels represent risk of a regional epidemic and outbreak, respectively, but no dengue transmission was detected in any of the tested areas with the exception of Jiaxiang. This finding suggested that the outbreak of local dengue cases was not only closely related with *Aedes* mosquitoes but also with the infection source (dengue-infected people) and susceptible populations. This association might also be driven by a multifactorial process that includes lack of emphasis and recognition, environmental changes, urbanization and absence of talent. Jiaxiang has a large airport with frequent personnel exchanges and is adjacent to Nanyang Lake, and the New Zhuzhao River and Cai River pass through the district. Therefore, the Jiaxiang district is rich in water and rice in early July, which can increase the number of small-sized water bodies. The district has a large garage overflown with waste tires and used bottles. All of these features are suitable for mosquito breeding and could increase mosquito populations in the environment. Young adults who make a living in metropolitan and foreign countries during the slack seasons of farming and return during the busy seasons (June, July and August) might be infected with dengue and transport the virus to the area of study. The “left-behind” children and elderly individuals are more likely to show a lack of knowledge of dengue, which might result in a notable escalation of the likelihood of contact between humans and mosquitoes, and therefore, an increase in the risk of dengue transmission.

Due to the combination of risk factors exposed above, a new dengue case is expected to occur in Jiaxiang and even in Shandong Province. Therefore, strengthening surveillance, community mobilization and education could help reduce the incidence of dengue.

Because larvae do not directly transmit disease and the density of larvae does not represent the adult density, we also monitored the density of adult mosquitoes using the HDN method. This method exhibits a clear advantage in protecting attractors and remains an efficient monitoring method in epidemic areas and during dengue outbreaks [31, 37]. Similar research was performed in Hangzhou [32], where local dengue cases were first reported in August 2017 and the disease spread rapidly. The biting rate from September to October 2017 obtained using the HDN method was 2.29–8.50 f/m/h. A biting rate of 2–11 f/m/h was obtained using the HDN method from October to November 2014 in Shenzhen [38], where an outbreak of dengue fever occurred in 2014. Previous studies have shown [11, 33] that adult mosquito sampling can provide more valuable data for studies of seasonal population trends and for assessing the risk of dengue outbreaks compared with that obtained through larval sampling. However, the results obtained from adult sampling are less reproducible, and the density of *Aedes* mosquito larvae in different years is affected by natural and social factors during monitoring. Natural factors [35] include the geographical location, quantity and size of breeding sites, meteorological factors [39] and quantity of animals, whereas social factors [40, 41] mainly comprise the control activities implemented by governments and non-government organizations and the quality of health conditions. Therefore, the larval and adult mosquito density should be monitored simultaneously, and targeted prevention and control measures should be taken to effectively reduce the density of *Aedes* mosquitoes and the risk of dengue transmission.

Previous studies [29, 42–44] only detected non-synonymous mutations in domain III (1532 and 1534) in *Ae. albopictus* from China. Therefore, we focused on these two sites and found I1532T, F1534L and F1534S mutations. The results obtained in this study were similar to those obtained in other studies: the F1534S mutant allele greatly enhances the *Vgsc* insensitivity to deltamethrin, and the F1534L allele does not increase the resistance of mosquitoes to deltamethrin [29, 44, 45]. However, the other results showed differences. In the present study, the I1532T mutation, which appeared only in the mosquito populations from Italy and Hainan Province, China, was detected [45], and a previous study in Hainan showed that this mutation was negatively associated with the deltamethrin resistance phenotype [42]. However, in the present study, the I1532T mutation was positively correlated with the deltamethrin resistance phenotype. The F1534S and I1532T mutations reduce sensitivity to pyrethroids in mosquitoes, but the inactivation mechanism is unknown. *kdr* mutations were found at other positions, including S989P and V1016G on *Vgsc*, in *Aedes* mosquitoes, and these mutations greatly increase the insensitivity to pyrethroids [30, 46–48]. In our study, the F1534C mutant allele, which is negatively correlated with pyrethroid resistance in southern China, was not found in this study area [49]. In addition, V1016G and V1016I mutation in IIS6 [29], which might be positively related to pyrethroid resistance, were not tested in our study. Thus, *kdr* mutations need further research.

## Conclusions

Although the local circulation of dengue fever in China has not been clearly established [50], imported cases of dengue fever will continue to be reported for a long time, particularly in economically developed, border and densely populated areas. In addition, the risk of dengue fever has expanded to the north and west in China [51]. The geographical area and number of people at risk have increased significantly. In Jining and even Shandong Province, which constitutes a high-latitude area at high risk for dengue fever outbreak, *Aedes* density surveillance, resistance monitoring and risk assessment should be strengthened to ensure the sustainable control of *Aedes* and thus the prevention and control of dengue transmission.

## Supplementary information

**Additional file 1: Table S1.** Mosquito sampling sites.

**Additional file 2: Table S2.** The BI value between 2017 and 2018.

**Additional file 3: Table S3**. The CI value between 2017 and 2018.

**Additional file 4: Table S4**. Biting rate of *Ae. albopictus* females collected per person per hour using the HDN method.

**Additional file 5: Table S5**. Genotypes at codons 1532 and 1534 in *Ae. albopictus.*

## Data Availability

All data generated or analysed during this study are included in this published article and its additional files.

## Abbreviations

*Vgsc*: voltage-gated sodium channel
*kdr*: knockdown resistance
BI: Breteau index
WHO: World Health Organization
CDC: Center for Disease Control and Prevention
